# Treatment results and prognostic factors of pediatric neuroblastoma: a retrospective study

**DOI:** 10.1186/1755-7682-3-37

**Published:** 2010-12-24

**Authors:** Mohamed I El-Sayed, Amany M Ali, Heba A Sayed, Eman M Zaky

**Affiliations:** 1 Department of Radiation Oncology, South Egypt Cancer Institute (SECI), Assiut University, Assiut, Egypt; 2 Department of Pediatric Oncology, South Egypt Cancer Institute (SECI), Assiut University, Assiut, Egypt; 3 Department of Clinical Pathology; South Egypt Cancer Institute (SECI), Assiut University, Assiut, Egypt

## Abstract

**Background:**

We conducted a retrospective analysis to investigate treatment results and prognostic factors of pediatric neuroblastoma patients.

**Methods:**

This retrospective study was carried out analyzing the medical records of patients with the pathological diagnosis of neuroblastoma seen at South Egypt Cancer Institute, Assiut University during the period from January 2001 and January 2010. After induction chemotherapy, response according to international neuoblastoma response criteria was assessed. Radiotherapy to patients with residual primary tumor was applied. Overall and event free survival (OAS and EFS) rates were estimated using Graphed prism program. The Log-rank test was used to examine differences in OAS and EFS rates. Cox-regression multivariate analysis was done to determine the independent prognostic factors affecting survival rates.

**Results:**

Fifty three cases were analyzed. The median follow-up duration was 32 months and ranged from 2 to 84 months. The 3-year OAS and EFS rates were 39.4% and 29.3% respectively. Poor prognostic factors included age >1 year of age, N-MYC amplification, and high risk group. The majority of patients (68%) presented in high risk group, where treatment outcome was poor, as only 21% of patients survived for 3 year.

**Conclusion:**

Multivariate analysis confirmed only the association between survival and risk group. However, in univariate analysis, local radiation therapy resulted in significant survival improvement. Therefore, radiotherapy should be given to patients with residual tumor evident after induction chemotherapy and surgery. Future attempts to improve OAS in high risk group patients with aggressive chemotherapy and bone marrow transplantation should be considered.

## Introduction

Neuroblastoma is the third most common malignancy in childhood and accounts for at least 15 percent of cancer-related deaths in children [1]. Despite concerted clinical and scientific efforts, prognoses of patients with high-risk neuroblastoma remain poor. About 30 to 35% of patients with high-risk disease who are older than 1 year survive more than 5 years [2,3].

Children with neuroblastoma exhibit marked variability in outcome when the disease is categorized by age, stage, and biologic characteristics [4,5]. Efforts to improve the outcome of patients with neuroblastoma have focused on identification of risk groups based on clinical and biologic variables as well as intensification of therapy for high-risk cases [6].

## Patients and Methods

### Study subjects

This retrospective study was carried out analyzing the medical records of patients with the pathological diagnosis of neuroblastoma seen at the in the Pediatric Oncology, Clinical pathology and Radiotherapy Departments, SECI, Assiut University during the period from January 2001 and January 2010. Informed consent was obtained for all patients and the treatment decision was approved by institutional review board at our center. Eligible patients had histologically confirmed, neuroblastoma, and were previously untreated. For each patient, evaluation was done by history and examination, routine laboratory investigations, and imaging studies in the form of CT scan with contrast for the local disease, and bone scan. Bone marrow aspirate and biopsy was done for all patients. Histopathologic diagnosis was obtained from presenting mass and for uncertain cases, immunohistochemistry by neuron specific enulase was done. Patients were staged by international neuroblastoma staging system [7]. N-MYC amplification was done for all patients, using FISH in paraffin-embedded tissue sections.

### Treatment Schedule

For patients with stage IVs; nine courses of doxorubicin (1 mg/kg) and cyclophosphamide (33 mg/kg) regimen (every 3 weeks), were given. For patients with stage III and IV disease; treatment included six courses of OPEC alternating with OJEC chemotherapy [table 1]. After the third and six courses, patients were evaluated for chemotherapy response by clinical examination, bone marrow aspirate and biopsy, CT scan of the local site and bone scan according to the initial presentation. Response according to international neuroblastoma response criteria [7] was assessed. Radiotherapy was given to patients with residual primary tumor, and for patients with cord compression as palliative treatment. CT planning was done for determination of target volume and critical structures. The radiation target volume included 1 cm margin around all sites of residual disease evident after induction chemotherapy and surgery. Radiotherapy was delivered using 6-MeV photon beams and antero-posterior/postero-anterior fields with customized blocks. A total dose of 24 Gy was given in 16 fractions [1.5 Gy per fraction]; 5 fractions per week. For younger children who needed anesthesia for immobilization, a total dose of 21 Gy was administered in hypofractionated schedule; 3 fractions [2.1 Gy per fraction] per week for 10 fractions. The clinical target volume had to be covered by the 95% isodose calculated for the reference point.

**Table 1:** Chemotherapy protocols.

OPEC regimen	Dose	Route and duration of administration	Timing

Vi Vincristine	1.5 mg/m2	i.v.	Day 1
Cisplatin	80 mg/m2	i.v. (6 hours infusion)	Day 2
Etoposide	200 mg/m2	i.v. (2 hours infusion)	Day 4
Cyclophosphamide	600 mg/m2	i.v. (2 hours infusion)	Day 1
Alternating every 3 weeks with **OJEC regimen**			
Vincristine	1.5 mg/m2	i.v.	Day 1
Carboplatin	500 mg/m2	i.v. (6 hour infusion)	Day 1
Etoposide	200 mg/m2	i.v. (2 hours infusion)	Day 1
Cyclophosphamide	600 mg/m2	i.v. (2 hours infusion)	Day 1

### After-Therapy Monitoring

After completion of therapy, patients were followed up regularly every 3 months for 1 year, every 6 months for the next 2 years, and annually thereafter. Follow-up examinations included physical examination, and routine laboratory studies. CT scans, and bone scan were done according to initial presentation. Assessment for treatment-related organ toxicity was done during treatment and after completion of therapy during follow up.

### Statistical Methods

The study cutoff point was January 2010. Overall survival (OAS) was defined as the interval from enrollment to the date of death from any cause or to last follow-up. Event free survival (EFS) was defined as the interval from enrollment of patients to the date of relapse, progression, or death from any cause or to the date of last follow-up. Overall and event free survival rates were estimated using Graphed prism program. The Log-rank test was used to examine differences in OAS and EFS rates. Cox-regression multivariate analysis was done to determine the independent prognostic factors affecting survival rates.

## Results

### Patients' Characteristics [Table 2]

**Table 2:** Patients' characteristics.

Variable	No	%
**1. Age at diagnosis**:		
a) <1 year	12	22.6
b) ≥ 1 year	41	77.4
**2. Sex**		
a) Males	35	18
b) Females	66	34
**3. Stage**		
a) III	13	24.6
b) IV	36	67.9
c) IVS	4	7.5
**4. Risk group**		
a) Low Risk	4	7.5
b) Intermediate Risk	13	24.6
c) High Risk	36	67.9
**5. Primary site**		
a) Adrenal glands	46	86.8
b) Paraspinal	4	7.5
c) Pelvis	2	3.8
d) Mediastinal	1	1.9
**6. Metastatic sites**		
a) No metastasis	13	24.5
b) Bone marrow	18	34
c) Bone marrow and bone	14	26.4
d) Bone marrow and brain	2	3.8
e) Bone marrow and lung	1	1.9
f) Bone marrow, bone and liver	1	1.9
g) Bone marrow, lung and liver	1	1.9
h) Bone	1	1.9
i) Liver	1	1.9
j) Subcutaneous nodules	1	1.9
**7. MYCN amplification**		
a) yes	11	20.8
b) no	42	79.2
		
**8. Local Radiotherapy administration**		
a) yes	25	47.2
b) no	28	52.8
**TOTAL**	53	100

The median age of patients was 2 years (range: 3 months - 13 years). Twelve patients (22.6%) were under one year of age, and 41 patients (77.4%) above 1 year (65%). Thirty five patients were male (66%) and 18 were females (34%). The majority of patients were ≥1 year of age (41 patients; 77.4%), males (35 patients; 66%), presented with stages III & IV (49 patients; 92.5%), and with suprarenal tumor (46 patients; 86.8%). Low risk group (n = 4, 7.5%) included patients with stage IVs disease. Intermediate risk group (n = 13, 24.6%) included patients of <1 year of age with III disease and non amplified N-MYC (n = 12) as well as one patient of <1 year of age with stage IV disease and non amplified N-MYC. High risk group (n = 36, 68%) included one patient of >1 year of age with stage III and amplified N-MYC, one patient <1 year of age with stage IV disease and amplified N-MYC and patients >1 year of age with stage IV disease (n = 34). Thirty six patients (67.9%) had BM infiltration, and 14 patients (26.4%) had positive bone scan. N-MYC amplification was detected in only 11 patients (20.8%). Adrenalectomy was done in 12 patients, adrenalectomy with nephrectomy in 2 patients, and pelvic mass excision in 2 patients. Radiotherapy to the site of residual primary tumor was given in 25 patients (47.2%), while palliative irradiation was given to 12 patients with painful bone metastases. The median follow-up from the date of enrollment was 32 months and ranged from 2 to 84 months.

### For patients <1 year of age (n = 12)

#### For patients with stage IVs (n = 4)

After nine courses of doxorubicin and cyclophosphamide regimen, these patients achieved CR.

#### For patients with stage III (n = 6)

After 3 courses of OPEC/OJEC regimen, all 6 patients achieved PR. After 3 additional chemotherapy courses, 4 patients underwent surgical resection and the other 2 patients were irresectable. All 6 patients had received local radiotherapy.

#### For patients with stage IV (n = 2)

These 2 patients achieved PR after 6 courses of OPEC/OJEC regimen. They underwent surgical resection, but with residual disease. They had received local radiation therapy.

### For patients ≥1 year of age (n = 41)

#### For patients with stage III (n = 7)

After 3 courses of OPEC/OJEC regimen, one patient died during chemotherapy at 2 months follow up, and 6 patients showed mixed response, but were irresectable. After additional 3 chemotherapy courses, they became resectable, underwent surgical resection, but with residual disease and received radiotherapy on residual tumor.

#### For patients with stage IV (n = 34)

After 3 courses of OPEC/OJEC regimen, 5 patients lost follow up, and 9 patients showed rapidly progressive disease with deteriorated general condition and eventually died; 2 of them died during chemotherapy and 7 shortly after it at follow up range of 4-8 months. For these 7 patients palliative radiotherapy was given to sites of painful bone metastases. Six patients had progressive disease and 14 showed mixed responses. After additional 3 chemotherapy courses, 6 patients with progressive disease showed stable disease, but were irresectable and received local radiotherapy. Four patients of those with mixed response showed stable disease and surgical resection was attempted. These 4 patients died in the early postoperative period (3 months). Ten patients of those with mixed response showed stable disease, but were irresectale. Five of these 10 patients showed deteriorated general condition, received palliative radiotherapy to sites of painful bone metastases, and eventually died (at a follow up range of 9-10 months). The remaining 5 patients were given local radiotherapy.

### Survival analysis [Table 3, 4 and Figure 1, 2, 3 and 4]

**Table 3:** Univariate analysis of prognostic factors affecting OAS and EFS rates.

P value	3-year EFS	P value	3-year OAS	factor
**1-Age**		**P = 0.0005**		**P = 0.0004**
1 year (12)>	80%	HR:0.26,95%CI;	80%	HR:0.26,95%CI;
≥1 years (41)	28.9%	0.12-0.55	16.2%	0.12-0.55
**2-Sex**		**P = 0.902**		**P = 0.84**
Males (35)	41.4%	HR:1.05,95%CI;	30.9%	HR: 1.09, 95%CI;
Females (18)	34.7%	0.502-2.184	28.1%	0.52 - 2.24
**3-Risk group**		**p < 0.0001**		**p < 0.0001**
Low Risk (4)	100%		100%	
Intermediate Risk (13)	74.6%		74.6%	
High Risk (36)	20.7%		6.1%	
**4-Primary site**		**P = 0.059**		**P = 0.23**
a) Adrenal glands (46)	32.7%	HR:2.44,95%CI;	27%	HR: 1.77, 95%CI;
b)Other sites (7)	71.4%	0.97-6.16	57.1%	0.697 - 4.530
				
**5-MYCN amplification**		**P = 0.0027**		**P < 0.0001**
yes (11)	8.3%	HR:4.58; 95%CI;	0	HR:10.64; 95%CI;
no (42)	49%	1.69 - 12.37	37.2%	3.47 - 82.63
**6-Local RT administration**		**p = 0.0078**		**p = 0.032**
Yes (25)	57.6%	HR:0.39; 95%CI;	42.5%	HR:0.47; 95%CI;
No (28)	23.4%	0.19 - 0.78	19.5%	0.24-0.94

**Table 4:** Results of the multivariate Cox regression analysis.

Factor	Significance	HR	95% CI
**OAS**			
Risk group	<0.0001	27.96	5.59 - 139.87
**PFS**			
Risk group	<0.0001	38.08	6.87 - 210.84

**Figure 1:**
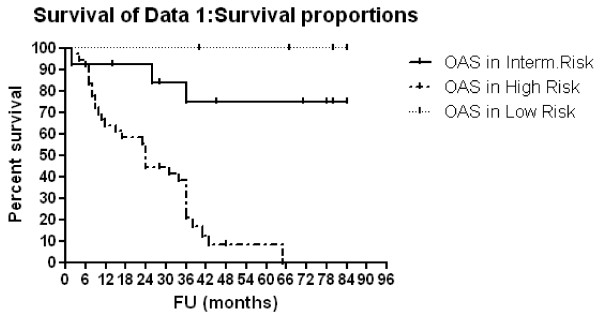
**OAS according to risk group**.

**Figure 2:**
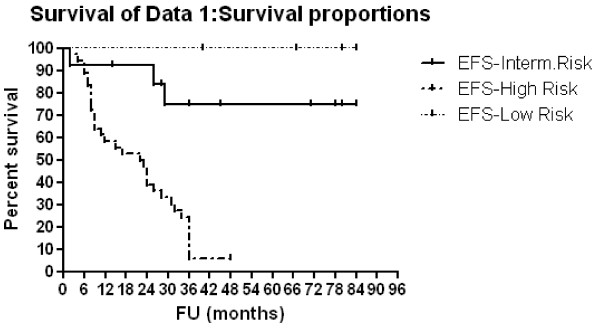
**EFS according to risk group**.

**Figure 3:**
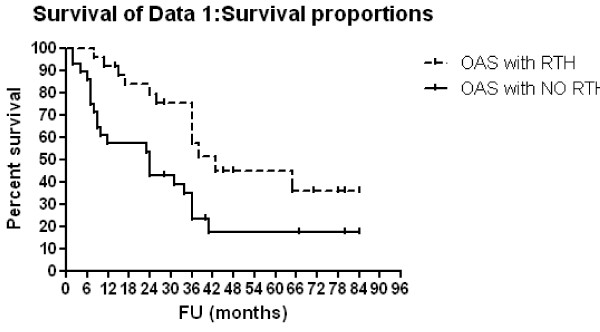
**OAS according to radiotherapy administration**.

**Figure 4:**
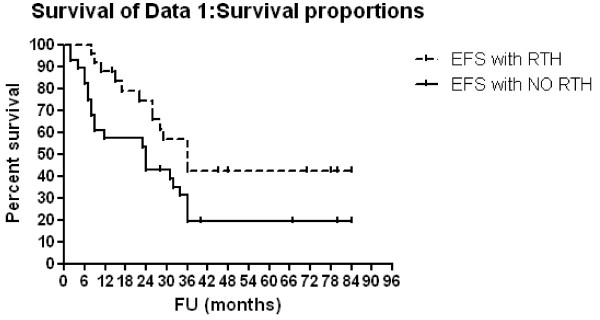
**EFS according to radiotherapy administration**.

Three-year OAS and EFS rates were 39.4% and 29.3% respectively, for all patients. Patients with <1 year of age showed 3-year OAS and EFS of 80% compared to 28.9% (p = 0.0005, HR: 0.26, 95% CI: 0.12-0.55) and 16.2% (p = 0.0004, HR: 0.26, 95% CI: 0.13-0.55) respectively, for older patients. For patients with low, intermediate and high risk diseases,3-year OAS rates were 100%, 74.6% and 20.7% respectively (p < 0.0001) and 3-year EFS rates were 100%, 74.6% and 6.1% respectively (p < 0.0001). Multivariate Cox regression analysis confirmed only the association between risk group and both OAS (p = < 0.0001, HR: 27.96, 95% CI: 5.59-139.87) and EFS (p = < 0.0001, HR: 38.08, 95% CI: 6.87-210.84) rates. Patients with N-MYC amplification showed 3-year OAS and EFS rates of 8.3% and 0% respectively compared to 49% (p = 0.0027, HR: 4.58, 95% CI: 1.69 - 12.37) and 37.2% (p < 0.0001, HR: 10.64, 95% CI: 3.47 - 32.63) respectively for those no MYCN amplification. For patients who were given local radiotherapy, 3-year OAS was 57.6% compared to 23.4% for those who were not given RT (p = 0.0078, HR: 0.39, 95% CI: 0.19-0.78) while the 3-year EFS rate was 42.5% for irradiated patients compared to 19.5% for non irradiated patients (p = 0.032, HR:0.47; 95%CI; 0.24-0.94).

### Acute Toxicity and Late Effects [Table 5]

**Table 5:** Treatment toxicity rates.

Toxicity	Grade III	
	
	No	(95% CI)%
Leucopenia	24	45.3 (31.88 - 58.68)
Thrombocytopenia	21	39.6 (26.45 - 52.79)
Infection	18	34 (21.21 - 46.71)
Increased liver enzymes	18	34 (21.21 - 46.71)
Nausea and vomiting	10	18.9 (8.34 - 29.4)
Ototoxicity	7	13.2 (4.09 - 22.33)
Increased serum creatinine	4	7.6 (0.44 - 14.66)

Therapy was well tolerated; where only two patients (of high risk group) died from septicemia due to febrile neutropenia. There was also one patient (of high risk group) developed chest infection after relapse. Chest X ray, CT chest and pulmonary functions tests revealed pulmonary fibrosis and this patient died in relapse. There is no late toxicity developed during the study period. Regarding grade III hematological toxicity, leucopenia occurred in 24 patients (45.3%), and thrombocytopenia in 21 patients (39.6%). Regarding grade III non hematological toxicity; infection in 18 patients, increased liver enzyme in 18 patients (34%), ototoxicity in 7 patients (13.2%) and increased serum creatinine in 4 patients (7.6%).

## Discussion

Most children with low risk neuroblastoma can be cured with surgery alone [8]. Most infants with disseminated disease have favorable outcomes following treatment with chemotherapy and surgery [4]. In contrast, the majority of children with high risk disease die from progressive disease despite intensive multimodality therapy. Current multimodality protocols for high-risk neuroblastoma patients have incorporated radiation to the primary disease site [9]. It was suggested that radiation to the primary tumor bed might benefit patients with gross residual disease at the time of radiation [10], with irradiation of active tumor residual instead of initial tumor extension [11].

In the current study, the median age of patients was 2 years and was confirmed by Papaioannou and McHugh [12] who stated that the most undifferentiated and aggressive neuroblastoma presents in young children with the median age ≤2 years. Compared with the current study, the reported studies, showed higher incidence of infants (45%) [13] and lower male to female ratio (1.3: 1) [14] and this may be explained by higher total number of patients (n = 500 - 594) in their studies.

Most of our patients presented with stage III & IV (92.5%). In the reported studies, it was found that the stages most frequently encountered at diagnosis were stages III and IV [15,16], and that 70% of the cases of children with NB presented with metastasis at diagnosis [17]. The distribution of our patients according to risk stratification (low risk in 7.5% and high risk in 68%) was different from that of Oberthuer et al. [18] (low risk in 50% and high risk in 40%) who used a gene expression-based classifier for neuroblastoma patients to reliably predicts courses of the disease. N-MYC amplification was detected in only 11 patients (21%) in the present study. This is lower than that found by Hass-Kogan et al., (27%) [10] and Schmid et al., (31%) [4], probably due to higher total number of patients (n = 134 - 539) in the reported studies [4,10].

The prognosis for neuroblastoma varies because of its peculiar biologic behavior [19]. The estimated OAS and EFS rates at 3 years for our patients were 39.4 and 29.3%, respectively. Three year OAS and EFS rates were significantly better in the 12 infants (80%) compared with the 41 children ≥1 year of age (29%; *P *= 0.0005 and 16.2%; *P *= 0.0004, respectively). This was confirmed by Evans and D'Angio, [20] who stated that, age younger than 1 year is a strongly favorable factor by itself. It was stated that OAS and EFS rates were significantly better in infants compared with children ≥1 year of age [21,22]. However, Schmidt et al [23], showed that EFS was 74% for the 12- to 18-month age group compared with 31% for those 18 to 24 months of age (*P *= .008).

The COG stratified patients into low, intermediate, or high risk categories based on prognostic features. Following a meeting in 2005 to review data obtained for 11054 patients treated between 1974 and 2002, age, stage at diagnosis, and N-MYC status were selected for initial risk grouping [24]. In the present study, for patients with low, intermediate and high risk diseases, 3-year OAS rates were 100%, 74.6% and 20.7% respectively (p < 0.0001) and 3-year EFS rates were 100%, 74.6% and 6.1% respectively (p < 0.0001). Multivariate analysis confirmed only the association between survival and risk group. In low risk patients,4-year EFS and OAS rates of 81% ± 4% and 98% ± 1.5%, respectively, were reported in previous CCG studies following treatment with surgery alone [8]. Our figures in low risk group (100%, 3-year OAS and EFS rates) were comparable to that reported by Bowman et al, [25] where the estimated 3-year OAS rate for patients with hyperdiploid tumors that lacked N-MYC amplification was 96%. These findings supported the reduction-in-therapy approach that was tested in the current COG low-risk study. The overall objective of the COG low-risk study was to preserve the excellent survival rate for patients with low-risk NB by using surgery as the primary treatment approach, thereby minimizing the risks of acute and long-term chemotherapy-related morbidity for the majority of these patients [26].

Survival rates in intermediate risk group (75%, 3-year OAS and EFS rates) in the present study was also comparable to that found in one of the reported study, where patients showed estimated 3-year EFS and OAS rates of 85% and 95% respectively [27].

Survival for high-risk children has improved modestly during the past 20 years, although cure rates remain low. Survival rates in high risk group (20.7%, 3-year OAS and 6% EFS rates) in the present study were very low. In the literatures, these patients had long-term survival of only 10 to 20 percent with combination chemotherapy, surgery, and local radiation therapy [1]. However, treatment approaches that used a combination of induction therapy, myloablative consolidation therapy with stem cell support, and biologic therapy had improved 5-year survival rates from less than 15% to 40% [24].

In the present study, patients with N-MYC amplification showed statistically significant lower 3-year OAS and EFS rates (8.3% and 0%, respectively) than OAS (49%, p = 0.0027) and EFS (37%, p < 0.0001) in patients with no N-MYC amplification. This was consistent with reported series [26].

Regarding radiotherapy, outcome analysis focused on EFS and OAS rather than local control rates, since local treatment must aim to improve the survival of each individual patient [19]. The NB97 radiotherapy approach (36 Gy to residual tissue on the local tumor site) did compensate the outcome disadvantage of incomplete response to induction therapy. The authors discussed that radiotherapy might be able to compensate a suspected disadvantage of residual tumor [11]. Our data supported this hypothesis, as there were statistically significant higher 3-year OAS and EFS rates (57.6% and 42.5%, respectively) in patients who were given local radiotherapy than OAS (23%, p = 0.0078) and EFS (19.5%, p = 0.032) in patients with no local radiotherapy. This was matched with Simon et al., [11] where 3-year EFS and OAS rates were statistically significant lower if residual primary tumor was not irradiated than those in irradiated patients. This was also confirmed by Castleberry et al., [28] who used chemotherapy with or without radiotherapy. Overall survival (73% vs 41%) was better in the group receiving radiation. Therefore, it is recommended to give local radiotherapy to the residual primary tumor site [10].

Grade III leucopenia occurred in 24 patients (45.3%), and thrombocytopenia in 21 patients (39.6%). In the reported studies, grade 3/4 hematologic toxicity was more common (66 - 71%) and may be due to longer duration of induction chemotherapy [4] or more intensive chemotherapy course [27]. Infection was detected in 18 patients (34%) in the current study, which was within the range (24 - 58%) found in the reported studies [27,29]. Seven patients (13.2%) developed ototoxicity and 4 (7.6%) developed renal impairment in the present study. These figures were comparable to those found in CCG-3881, where renal and ototoxicity greater than grade 2 occurred in 3-6% of patients [4,29].

## Conclusion

The majority of patients (68%) presented in high risk group, where treatment outcome was poor. Multivariate analysis confirmed only the association between survival and risk group. However, in univariate analysis, there were statistically significant higher 3-year OAS and 3-year EFS rates in patients who were given local radiotherapy compared to survival rates in non irradiated patients. Therefore, radiotherapy should be given to patients with residual tumor evident after induction chemotherapy and surgery. Future attempts to improve OAS in high risk group patients with aggressive chemotherapy and bone marrow transplantation should be considered.

## Conflict of interests

The authors declare that they have no competing interests.

## Authors' contributions

MIE carried out radiation therapy administration, follow up, statistical analysis, general coordination, drafting of the manuscript and writing of the final manuscript. AMA, and HAS carried out collection of patients' data, patient diagnosis, chemotherapy administration and follow up. EMZ identified N-MYC status using FISH technique. All authors have read and approved the final manuscript.

## References

[B1] Matthay KK Neuroblastoma: A clinical challenge and biologic puzzle CA Cancer J Clin 1995 45 179 192 10.3322/canjclin.45.3.179 7743421

[B2] Matthay KK Neuroblastoma: Biology and therapy Oncology 1997 11 1857 1866 9436190

[B3] Berthold F Hero B Kremens B Long-term results and risk profiles of patients in five consecutive trials (1979-1997) with stage 4 neuroblastoma over 1 year of age Cancer Lett 2003 197 11 7 10.1016/S0304-3835(03)00076-4 12880954

[B4] Schmidt ML Lukens JN Seeger RC Biologic factors determine prognosis in infants with stage IV neuroblastoma: A prospective Children's Cancer Group study J Clin Oncol 2000 18 1260 1268 10715296 10.1200/JCO.2000.18.6.1260

[B5] Goto S Umehara S Gerbing RB Histopathology (International Neuroblastoma Pathology Classification) and MYCN status in patients with peripheral neuroblastic tumors: A report from the Children's Cancer Group Cancer 2001 92 2699 2708 10.1002/1097-0142(20011115)92:10<2699::AID-CNCR1624>3.0.CO;2-A 11745206

[B6] Matthay KK Intensification of therapy using hematopoietic stem-cell support for high-risk neuroblastoma Pediatr Transplant 1999 3 72 77 10.1034/j.1399-3046.1999.00070.x 10587975

[B7] Brodeur GM Pritchard J Berthold F Revisions of the international criteria for neuroblastoma diagnosis, staging, and response to treatment J Clin Oncol 1993 11 1466 1477 8336186 10.1200/JCO.1993.11.8.1466

[B8] Perez CA Matthay KK Atkinson JB Biologic variables in the outcome of stages I and II neuroblastoma treated with surgery as primary therapy: a Children's Cancer Group study J Clin Oncol 2000 18 18 26 10623689 10.1200/JCO.2000.18.1.18

[B9] Matthay KK Villablanca JG Seeger RC Treatment of high-risk neuroblastoma with intensive chemotherapy, radiotherapy, autologous bone marrow transplantation, and 13-cis-retinoic acid N Engl J Med 1999 341 1165 1173 10.1056/NEJM199910143411601 10519894

[B10] Haas-Kogan DA Swift PS Selch M Haase GM Seeger RC Gerbing RB Stram DO Matthay KK Imact of radiotherapy for high risk neuroblastoma: A Children's Cancer Group Study Int J Radiation Oncology Biol Phys 2003 56 1 28 39 10.1016/S0360-3016(02)04506-6 12694821

[B11] Simon T Hero B Bongartz R Schmidt M Müller RP Berthold F Intensified external-beam radiation therapy improves the outcome of stage 4 neuroblastoma in children >1 year with residual local disease Strahlenther Onkol 2006 182 389 94 10.1007/s00066-006-1498-8 16826357

[B12] Papaioannou G McHugh K Neuroblastoma in childhood: review and radiological findings Cancer Imaging 2005 5 1 116 127 10.1102/1470-7330.2005.0104 16305949 PMC1665241

[B13] Urayama KY Von Behren J Reynolds P Birth Characteristics and Risk of Neuroblastoma in Young Children American Journal of Epidemiology 2007 165 5 486 95 10.1093/aje/kwk041 17164463

[B14] Aydn GB Kutluk MT Yalçn B Büyükpamukçu M Kale G Varan A Akyüz C Senocak ME Büyükpamukçu N Neuroblastoma in Turkish children: experience of a single center J Pediatr Hematol Oncol 2009 31 7 471 80 10.1097/MPH.0b013e3181a6dea4 19564739

[B15] Schüz J Kaletsch U Meinert R Kaatsch P Spix C Michaelis J Risk factors for neuroblastoma at different stages of disease. Results from a population-based case-control study in Germany J Clin Epidemiology 2001 54 702 709 10.1016/s0895-4356(00)00339-5 11438411

[B16] Juárez-Ocaña S Palma-Padilla V González-Miranda G Siordia-Reyes AG López-Aguilar E Aguilar-Martínez M Mejía-Aranguré JM Carreón-Cruz R Rendón-Macías ME Fajardo-Gutiérrez A Epidemiological and some clinical characteristics of neuroblastoma in Mexican children (1996-2005) BMC Cancer 2009 9 266 19650918 10.1186/1471-2407-9-266 PMC2729776

[B17] Papaioannou G McHugh K Neuroblastoma in childhood: review and radiological findings Cancer Imaging 2005 5 1 116 127 10.1102/1470-7330.2005.0104 16305949 PMC1665241

[B18] Oberthuer A Berthold F Warnat P Hero B Kahlert Y Spitz R Ernestus K König R Haas S Eils R Schwab M Brors B Westermann F Fischer M Customized Oligonucleotide Microarray Gene Expression-Based Classification of Neuroblastoma Patients Outperforms Current Clinical Risk Stratification Journal of Clinical Oncology 2006 24 31 5070 5078 10.1200/JCO.2006.06.1879 17075126

[B19] Kusumakumary P Ajithkumar TV Ratheesan K Chellam VG Nair MK Pattern and outcome of neuroblastoma: A 10 year study Indian Pediatrics 1998 35 223 229 9707875

[B20] Evans AE D'Angio GJ Age at Diagnosis and Prognosis in Children With Neuroblastoma Journal of Clinical Oncology 2005 23 27 6443 6444 10.1200/JCO.2005.05.005 16116157

[B21] Garaventa A Boni L Lo Piccolo MS Tonini GP Gambini C Mancini A Tonegatti L Carli M di Montezemolo LC Di Cataldo A Casale F Mazzocco K Cecchetto G Rizzo A De Bernardi B Localized unresectable neuroblastoma: results of treatment based on clinical prognostic factors Annals of Oncology 2002 13 6 956 964 10.1093/annonc/mdf165 12123342

[B22] London WB Castleberry RP Matthay KK Look AT Seeger RC Shimada H Thorner P Brodeur G Maris JM Reynolds CP Cohn SL Evidence for an Age Cutoff Greater Than 365 Days for Neuroblastoma Risk Group Stratification in the Children's Oncology Group Journal of Clinical Oncology 2005 23 27 6443 6444 10.1200/JCO.2005.05.571 16116153

[B23] Schmidt ML Lal A Seeger R Favorable prognosis for patients 12 to 18 months of age with stage 4 nonamplified MYCN neuroblastoma: A Children's Cancer Group Study J Clin Oncol 2005 23 6474 6480 10.1200/JCO.2005.05.183 16116154

[B24] Shusterman S George RE Orkin SH, Fisher DE, Look AT, Lux SE, Ginsburg D, Nathan DG Neuroblastoma 2009 Chapter 14 Oncology of infancy and childhood 509 540

[B25] Bowman LC Castleberry RP Cantor A Genetic staging of unresectable or metastatic neuroblastoma in infants: a Pediatric Oncology Group study J Natl Cancer Inst 1997 89 373 380 10.1093/jnci/89.5.373 9060959

[B26] Weinstein JL Katzenstein HM Cohn SL Advances in the Diagnosis and Treatment of Neuroblastoma The Oncologist 2003 8 3 278 292 10.1634/theoncologist.8-3-278 12773750

[B27] Bagatell R Rumcheva P London WB Cohn SL Look AT Brodeur GM Frantz C Joshi V Thorner P Rao PV Castleberry R Laura C Bowman LC Outcomes of Children With Intermediate-Risk Neuroblastoma After Treatment Stratified by MYCN Status and Tumor Cell Ploidy Journal of Clinical Oncology 2005 23 34 8819 8827 10.1200/JCO.2004.00.2931 16314642

[B28] Castleberry RP Kun LE Shuster JJ Radiotherapy improves the outlook for patients older than 1 year with Pediatric Oncology Group stage C neuroblastoma J Clin Oncol 1991 9 789 95 2016621 10.1200/JCO.1991.9.5.789

[B29] Kreissman SG Villablanca JG Diller L London WB Maris JM Park JR Reynolds CP von Allmen D Cohn SL Matthay KK Response and toxicity to a dose-intensive multi-agent chemotherapy induction regimen for high risk neuroblastoma (HR-NB): A Children's Oncology Group (COG A3973) study Journal of Clinical Oncology, 2007 ASCO Annual Meeting Proceedings Part I 2007 25 18S 9505

